# The gut mycobiome of the Human Microbiome Project healthy cohort

**DOI:** 10.1186/s40168-017-0373-4

**Published:** 2017-11-25

**Authors:** Andrea K. Nash, Thomas A. Auchtung, Matthew C. Wong, Daniel P. Smith, Jonathan R. Gesell, Matthew C. Ross, Christopher J. Stewart, Ginger A. Metcalf, Donna M. Muzny, Richard A. Gibbs, Nadim J. Ajami, Joseph F. Petrosino

**Affiliations:** 1 Alkek Center for Metagenomics and Microbiome Research, Department of Molecular Virology and Microbiology, Baylor College of Medicine, Houston, TX USA; 2 Human Genome Sequencing Center, Baylor College of Medicine, Houston, TX USA

**Keywords:** Fungi, Microbiota, Microbiome, Fungal microbiome, Fecal microbiome, HMP, ITS2, There was a high degree o Spacer, Metagenomics

## Abstract

**Background:**

Most studies describing the human gut microbiome in healthy and diseased states have emphasized the bacterial component, but the fungal microbiome (i.e., the mycobiome) is beginning to gain recognition as a fundamental part of our microbiome. To date, human gut mycobiome studies have primarily been disease centric or in small cohorts of healthy individuals. To contribute to existing knowledge of the human mycobiome, we investigated the gut mycobiome of the Human Microbiome Project (HMP) cohort by sequencing the Internal Transcribed Spacer 2 (ITS2) region as well as the 18S rRNA gene.

**Results:**

Three hundred seventeen HMP stool samples were analyzed by ITS2 sequencing. Fecal fungal diversity was significantly lower in comparison to bacterial diversity. Yeast dominated the samples, comprising eight of the top 15 most abundant genera. Specifically, fungal communities were characterized by a high prevalence of *Saccharomyces*, *Malassezia*, and *Candida*, with *S. cerevisiae*, *M. restricta*, and *C. albicans* operational taxonomic units (OTUs) present in 96.8, 88.3, and 80.8% of samples, respectively. There was a high degree of inter- and intra-volunteer variability in fungal communities. However, *S. cerevisiae*, *M. restricta*, and *C. albicans* OTUs were found in 92.2, 78.3, and 63.6% of volunteers, respectively, in all samples donated over an approximately 1-year period. Metagenomic and 18S rRNA gene sequencing data agreed with ITS2 results; however, ITS2 sequencing provided greater resolution of the relatively low abundance mycobiome constituents.

**Conclusions:**

Compared to bacterial communities, the human gut mycobiome is low in diversity and dominated by yeast including *Saccharomyces*, *Malassezia*, and *Candida*. Both inter- and intra-volunteer variability in the HMP cohort were high, revealing that unlike bacterial communities, an individual’s mycobiome is no more similar to itself over time than to another person’s. Nonetheless, several fungal species persisted across a majority of samples, evidence that a core gut mycobiome may exist. ITS2 sequencing data provided greater resolution of the mycobiome membership compared to metagenomic and 18S rRNA gene sequencing data, suggesting that it is a more sensitive method for studying the mycobiome of stool samples.

**Electronic supplementary material:**

The online version of this article (10.1186/s40168-017-0373-4) contains supplementary material, which is available to authorized users.

## Background

Fungi are ubiquitous in our environment and are known to participate in natural and industrial processes including production of antibiotics, bread, cheese, and alcoholic beverages; decomposing natural debris; and providing nutrients to plants in soil. Of the estimated 5.1 million different species of fungi in the world, only around 300 cause disease regularly in humans [1, 2]. These relatively few fungi are responsible for millions of infections each year, from superficial infections of the skin and nails, to invasive infections of the lungs, blood, and brain [3]. However, with the high prevalence of fungi in the environment, it is not surprising that fungi are also found on and in our bodies as constituents of the human microbiome. The fungal microbiome, known as the mycobiome, is an understudied component of the human microbiome. Although the mycobiota make up a small proportion of the entire human microbiome [4], culture-independent methods utilizing high-throughput sequencing techniques have allowed scientists to begin to uncover the identity of our fungal commensals and determine their role in human health and disease.

Fungi have been detected in the guts of several mammals, including humans, mice, rats, pigs, and many ruminant and non-ruminant herbivores [5–7]. Characterization of C57BL/6 mice feces revealed greater than 97% of fungal sequences belonged to only 10 fungal species, identifying *Candida tropicalis* and *Saccharomyces cerevisiae* as the most abundant commensal fungi [5]. In humans, fungi have been found to colonize the gut shortly after birth [8]. In a study investigating correlations of archaea and fungi with diet, volunteers had an abundance of *Candida* and *Saccharomyces* species in their stool, with high *Candida* abundance associated with recent consumption of carbohydrates [9]. Fungi have been implicated in the exacerbation of several human diseases, including inflammatory bowel disease, graft versus host disease, Hirschsprung-associated enterocolitis, colorectal cancer, and advanced progression of hepatitis B virus infections [5, 10–15]. The confirmed presence of fungi as a part of the human microbiome, as well as their potential role as contributors to health and disease, highlight the need to characterize the healthy human mycobiome more deeply. Knowledge of a healthy mycobiome will aid in research identifying disease-contributing fungal species and better define fungal-bacterial relationships that are important for health.

One of the initial goals of the Human Microbiome Project (HMP) was to characterize the “healthy” human microbiome as a baseline for reference and comparison studies [16]. Microbial communities in HMP healthy donor stool samples were largely comprised of bacteria from the Bacteroidetes and Firmicutes phyla, but varied greatly between volunteers [17]. Although core operational taxonomic units (OTUs) were identified in HMP donor stool, the relative abundance of these core OTUs were found to vary nearly 5000-fold [18]. This suggests that what constitutes a healthy gut microbiome can be very different among individuals. However, the mycobiome was not investigated in initial HMP studies.

Using DNA previously extracted from longitudinally collected stool samples (two to three samples per volunteer, collected over an approximately 1-year period) from HMP volunteers recruited at Baylor College of Medicine (Houston, TX), we characterized the “healthy” human gut mycobiome. Internal Transcribed Spacer 2 (ITS2) sequencing confirmed that fungal diversity in the gut is low. *Saccharomyces* was found to be the most abundant fungal genus in healthy human stool, followed by *Malassezia* and *Candida*. These three genera were present in at least one sample from nearly every volunteer in this study, although the mycobiome was highly variable within and between individuals. Sequencing of the 18S rRNA gene revealed similar results to the ITS2 sequencing, but included the addition of the non-fungal microbial eukaryote (microeukaryote) *Blastocystis* as a prominent eukaryotic member of the gut microbiome. Additionally, fungi identified in metagenomic sequences from HMP stool samples agreed with the ITS2 sequencing results; however, deeper metagenomic sequencing is likely required to fully survey the fungal constituents of the gut. It is important to understand what constitutes a healthy human gut mycobiome as this allows for further understanding of fungal-bacterial and fungal-host interactions, which may contribute to human health and disease.

## Results

### Fungal diversity and composition in healthy human stool

To investigate gut fungal diversity and composition, a total of 333 HMP stool sample microbial DNA extractions were retrieved and underwent ITS2 amplification and sequencing. After rarefaction, the number of samples analyzed was reduced to 317 (from 147 volunteers), with each sample normalized to 1954 sequences. Missing taxonomic information in databases resulted in many fungal OTUs being classified as “Fungi sp.” These OTUs constituted 17% of the total OTUs. Altogether, 701 fungal OTUs were detected in the sample set, capturing 247 named genera.

**Fig. 1:**
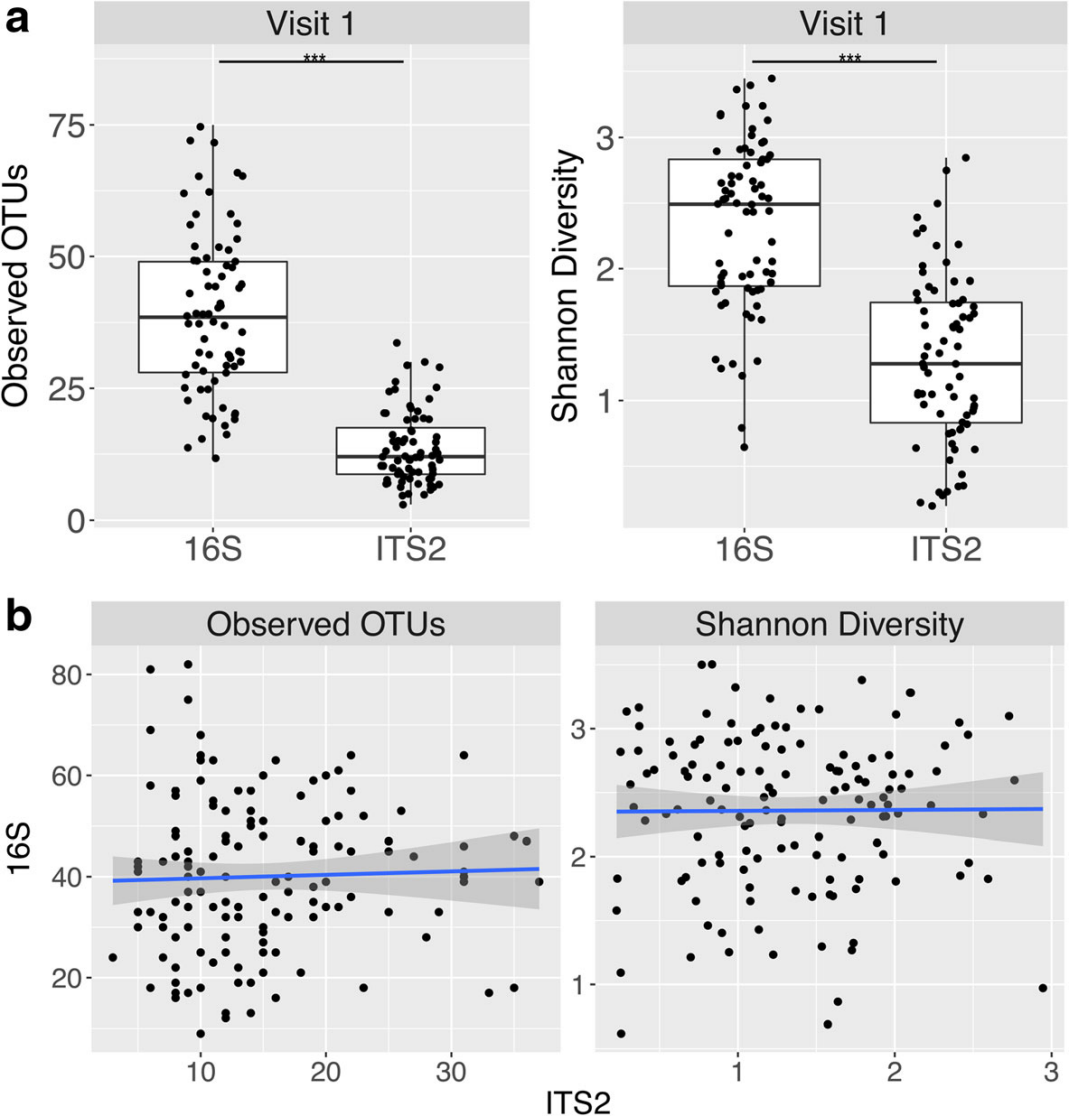
Fungal and bacteria alpha diversity. **a** Observed OTUs and Shannon diversity index values of HMP samples with both 16S rRNA gene and ITS2 sequencing data compared. Only visit 1 samples are shown for statistical purposes. Visit 2 and visit 3 comparisons showed similar results. For statistical analysis, only samples with both ITS2 and 16S rRNA gene sequencing data were used. ***P < 0.0001 for both observed OTUs and Shannon diversity index (Mann-Whitney test). **b** Associations between fungal (ITS2) and bacterial (16S) alpha diversity (observed OTUs and Shannon diversity index values) for a given sample. Shaded gray region represents 95% confidence intervals. Linear regression analysis: *P* = 0.693 for observed OTUs and *P* = 0.929 for Shannon diversity. Only samples with ITS2 and 16S rRNA gene sequencing data are plotted and analyzed

Observed OTUs within samples ranged from 2 to 92 (Table 1). The Shannon diversity index, which measures evenness and richness of communities within a sample, varied between 0.004 and 2.94, indicating low alpha diversity for most samples (Table 1). There was a significant difference between bacterial and fungal communities in both the number of observed OTUs and the Shannon diversity index values (Fig. 1a), supporting previous studies suggesting fungal diversity is lower than that of bacteria in a healthy human gut [19, 20]. No associations were observed between bacterial and fungal alpha diversity values as assessed by linear regression (Fig. 1).

**Table 1:** Alpha Diversity of fungal communities in HMP stool samples

	Observed OTUs	Shannon diversity index
Mean	14	1.27
Median	12	1.24
Minimum	2	0.004
Maximum	92	2.94

**Fig. 2:**
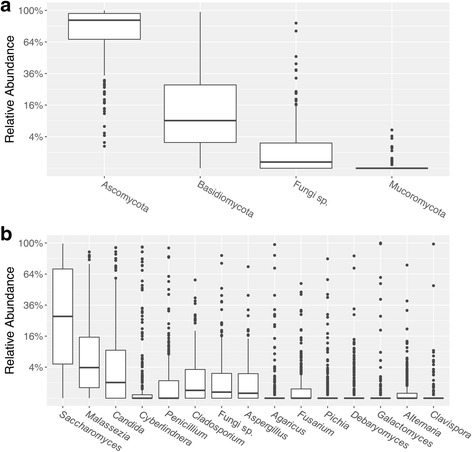
Relative abundance of fungi at the **a** phylum level and **b** genus level. **a** Relative abundance of fungal phyla in each sample. “Fungi sp.” here represents unknown/unidentified fungal phylum. **b** Relative abundance of fungal genera in each sample. “Fungi sp.” here represents unknown/unidentified fungal genus

Samples primarily consisted of fungi from the Ascomycota and Basidiomycota phyla, with Ascomycota being the most abundant phylum represented (Fig. 2a). The dominance of these phyla has been reported for other parts of the human body, including the skin, vagina, and oral cavity [21–24], suggesting that these phyla may be well-suited for life on mammalian hosts. *Saccharomyces* was the most abundant genus among all samples, followed by *Malassezia* and *Candida* (Fig. 2b). Overall, genera that include yeast species, including the three listed above as well as *Cyberlindnera*, *Pichia*, *Debaryomyces*, *Galactomyces,* and *Clavispora*, comprise eight of the 15 most abundant genera in the samples (Fig. 2b).

### Variability of the mycobiome

**Fig. 3:**
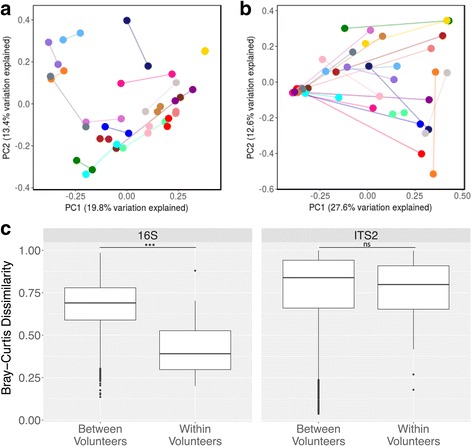
Variability of the mycobiome. **a** Bacterial (16S) and **b** Fungal (ITS2) Bray-Curtis dissimilarity shown on principal coordinates analysis (PCoA) plots for a subset of volunteers (20 volunteers, randomly chosen, subsetted for clarity). Samples are colored by volunteer, and each volunteer was assigned the same color in both **a** and **b**. Lines connect samples donated by the same volunteer. **c** Pairwise comparisons of Bray-Curtis dissimilarity values between samples donated by the same volunteer (within volunteers) and between samples donated by different volunteers (between volunteers) for 16S rRNA gene and ITS2 sequencing data. Bray-Curtis dissimilarity values range from 0 to 1, with 0 being the least dissimilar and 1 being the most dissimilar. ***P < 0.0001; ns: not significant

We sought to determine the variability between and within HMP volunteers’ gut fungal communities. In the original HMP study, the within-volunteer bacterial beta diversity measured between consecutive samples was lower (i.e., greater temporal similarity) compared to samples donated by other volunteers. That is, variation observed within a volunteer over time was lower than between-volunteer variation. This was true for all major body sites sampled [17]. In order to investigate whether within-volunteer fungal community diversity in the gut was lower than between-volunteer diversity as observed for bacterial communities, we measured variability using the Bray-Curtis dissimilarity metric. Ordination by principal coordinates analysis (PCoA) of bacterial (Fig. 3a) and fungal (Fig. 3b) communities reveals that HMP volunteers show more similar fecal bacterial community structure than fecal fungal community structure over time. Pairwise comparisons of Bray-Curtis dissimilarity values between longitudinal samples donated by the same volunteer and between samples donated by different volunteers for the 16S rRNA gene and ITS2 sequencing data were performed. The results reveal that, unlike bacterial communities, fungal communities exhibit high intra- and inter-volunteer dissimilarity (i.e., Bray-Curtis dissimilarity approaching 1.0) (Fig. 3 **c**). This indicates that while longitudinal samples of one individual’s fecal bacterial microbiome are more similar to each other than those of another individual, this does not appear to be the case for the fecal mycobiome.

### Stability of the mycobiome

To investigate the stability of the mycobiome, we measured the recurrence of fungal OTUs across all samples, as well as across each volunteers’ longitudinal samples. Despite the high degree of variability in the fungal communities of healthy human stool, there were several fungal taxa detected in a large proportion of HMP samples. *S. cerevisiae*, *M. restricta*, and *C. albicans* OTUs were present in 96.8, 88.3, and 80.8% of samples, respectively (Table 2). Additionally, longitudinal sampling of these volunteers allowed us to identify OTUs present at all visits of each volunteer. Excluding volunteers with only one sampling time point, *S. cerevisiae*, *M. restricta*, and *C. albicans* were detected at all visits in 92.2, 78.3, and 63.6% of volunteers, respectively (*n* = 129; Table 2). Although we observed great variability in the gut mycobiome among healthy volunteers, these three fungal species present in a majority of longitudinally collected samples suggest they may be resident commensals in the human gastrointestinal tract and part of our core gut mycobiome. However, we cannot rule out the possibility that consistent detection of these fungi in stool may indicate regular exposure to these organisms through environmental contact or diet.

**Table 2:** The prevalence of OTUs within samples and volunteers

OTU	% of samples with OTU (*n* = 317)	% of volunteers with OTU at all time points (*n* = 129)
*Saccharomyces cerevisiae*	96.8	92.2
*Malassezia restricta*	88.3	78.3
*Candida albicans*	80.8	63.6
*Candida sake*	62.1	40.3
*Cyberlindnera jadinii*	62.1	40.3
*Cladosporium* sp.	59.3	34.9
*Penicillium* sp.	46.7	24.0
*Galactomyces candidum*	46.1	38.0
*Malassezia globosa*	36.0	12.4
*Agaricus bisporus*	35.0	17.1

### Associations with host phenotype

To determine whether the mycobiome was associated with any host phenotypes, we utilized clinical metadata collected on HMP volunteers. These metadata include age, gender, BMI, race/ethnicity, tobacco use, insurance status, and more (for full list, see Additional file 1). Although the HMP consortium was able to identify modest associations between host phenotype and bacterial communities [17], based on EnvFit analysis [25], no significant covariate was associated with mycobiome profiles. Our data suggest, in line with conclusions from the HMP study, that the majority of variation in the human microbiome is not explained well by available phenotypic metadata and that other factors such as diet, environment, daily cycles, and host genetics may play a larger role in influencing the human gut mycobiome.

### Correlations between taxa

**Fig. 4:**
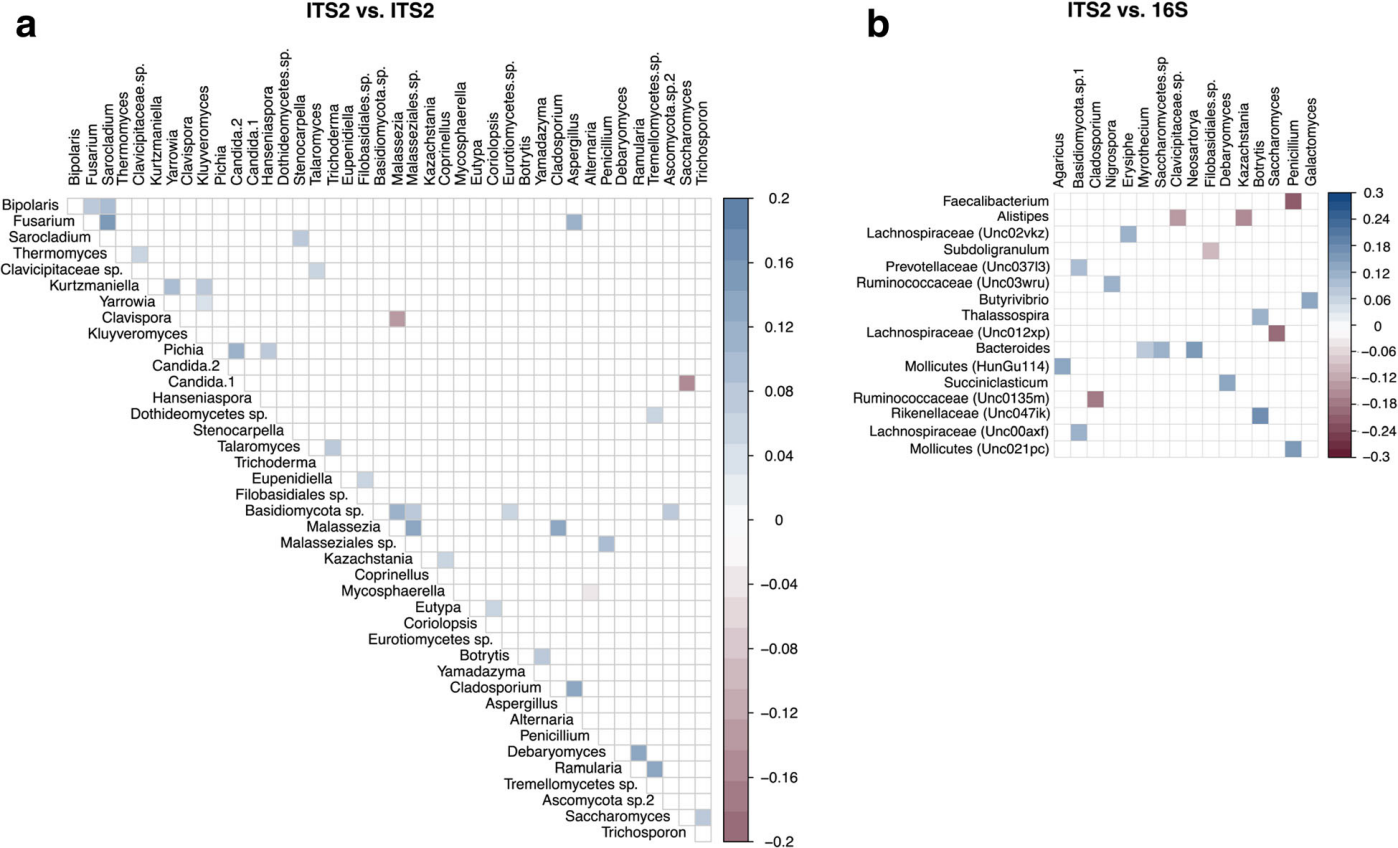
Correlations occurring between fungal taxa (ITS2) and **a** fungal taxa (ITS2) or **b** bacterial taxa (16S). Red squares represent significant (*P* < 0.05 after FDR adjustment) negative correlations. Blue squares represent significant (*P* < 0.05 after FDR adjustment) positive correlations. Darker colors represent stronger correlations. Non-significant correlations are not shown

We investigated taxa correlations by combining available 16S rRNA gene and ITS2 sequencing data from the same samples. Both fungal-fungal relationships and fungal-bacterial relationships were interrogated using SparCC [26]. Analysis of HMP fecal ITS2 sequencing data revealed the strongest positive correlation occurred between *Sarocladium* and *Fusarium*, while *Candida* and *Saccharomyces* exhibited the strongest negative correlation (Fig. 4a). Comparing abundances of fungal and bacterial genera using SparCC revealed both positive and negative correlations between taxa in the two domains (Fig. 4b). *Rikenellaceae* and *Botrytis* showed the strongest inter-domain positive correlation, and *Penicillium* and *Faecalibacterium* exhibited the strongest negative correlation. The biological relevance of these correlations remains unknown, but identification of relationships between fungal and bacterial taxa within a healthy human gut may reveal interactions that inform future studies seeking to modulate the relative abundances of certain taxa in the gut microbiome (e.g., through the use of fungi or fungal metabolites that impact targeted bacterial species).

### 18S rRNA gene sequencing

To determine whether the ITS2 primers were appropriately capturing the majority of the mycobiome taxa, a region of the 18S rRNA gene was amplified from 44 Baylor College of Medicine volunteers who had three sample collections. The 18S rRNA gene is more conserved and typically cannot resolve taxonomy as well as the ITS region, but provides an independent measure of fungal diversity that can identify biases in ITS2 analysis. The 18S rRNA gene-specific primers used by the Earth Microbiome Project (139f/EukBr) and Parfrey et al. (515f/1119r) [27] amplify a significant number of bacterial 16S rRNA genes. Therefore, to reduce the unwanted 16S rRNA gene signal, we designed new primers that would capture eukaryotic diversity as broadly as possible, while better discriminating against bacterial 16S rRNA gene targets. Utilizing two studies that identified the best regions for generating 18S rRNA gene primers [28, 29] and an alignment of sequences from representatives of every known microeukaryotic group, we designed the 1152F and 1428R (*S. cerevisiae* numbering) primers. These primers amplify the 18S rRNA gene V6/V7 region, which is of an appropriate size for Illumina MiSeq sequencing. Primer 1152F had at most 1 mismatch with all examined representative microeukaryotes, while 1428R had at most 2 mismatches. The region internal to the primers varied from 211 base pairs (Microsporidia) to 380 base pairs (Acanthamoeba). Since PCR and sequencing with a 400-base pair control showed a similar number of reads as a 271-base pair control, we were confident that there was not a dramatic bias against organisms with large V6/V7 18S rRNA gene regions.

18S rRNA gene sequencing of HMP samples yielded a mean of 17,189 reads/sample. While no reads mapped to bacteria, just 16% mapped to fungi, with the remaining reads mapping to mammals (mean 57% reads/sample), Stramenopiles (13%), plants (13%), non-mammalian animals (0.2%), Intramacronucleata (0.02%), and Amoebozoa (0.001%).The 18S rRNA gene sequencing of HMP stool DNA was different from the ITS2 sequencing data in its detection of the animals Mammalia (presumably mostly food- or host-derived), Aves (bird), Teleostei (fish), Ostreoida (oyster), Heterobranchia (snail), Diptera (fly), Acari (mites/ticks), and Collembola (springtail), the microeukaryotes *Blastocystis* (Stramenopiles), *Entamoeba* (Amoebozoa), Chromulinaceae sp. (chrysophyte flagellate), and Colpodea (ciliate). Only one fungus, the basidiomycete *Tritirachium*, was detected by 18S rRNA gene sequencing but not ITS2 sequencing. Notably, 18S rRNA gene sequencing results revealed the presence of the non-fungal microeukaryote *Blastocystis* in HMP stool samples*.* Multiple *Blastocystis* subtypes (ST) were detected: 19 samples from 11 volunteers had at least 10 sequences of *Blastocystis*, dominating 12 of those samples with > 99.9% of their 18S rRNA gene sequences (Additional file 2: Table S1). Volunteers with detectable *Blastocystis* had increased bacterial diversity, but no significant difference in their fungal diversity was observed (Additional file 2: Figure S1). Filtering out host and plant sequences from 18S rRNA gene sequencing data left 37 volunteers (66 samples) with at least 100 sequences. The four most abundant fungi detected were the same as observed using ITS2 primers: *Saccharomyces*, *Malassezia*, *Candida*, and *Cyberlindnera.*

### Fungi in metagenomic sequences

We sought to investigate whether fungi could be detected in HMP fecal metagenomic sequencing data. The HMP included relatively deep (targeting 10 gigabases per sample) whole genome shotgun (WGS) sequencing on stool samples to investigate metabolic pathways encoded by fecal bacteria [16]. We mined these metagenomic sequences for reads that map to fungal genomes. Of the > 27 billion metagenomic sequences generated, approximately 0.01% aligned to fungal genomes. Mapped fungal sequences in each sample supported the ITS2 sequencing data, finding *Saccharomyces* spp., *Malassezia* spp., and *Candida* spp. among the most abundant fungi (Table 3; Additional file 3: Tables S2 and S3). Additionally, species in these genera were detected in a large number of samples and volunteers, though not as prevalently as what was identified in the ITS2 sequencing data. This is likely due to insufficient WGS sequencing depth given the extremely low abundance of fungi in stool, in which higher abundance microbes like bacteria comprise the majority of metagenomic sequencing data. Greater WGS sequencing depth, as well as more complete fungal genomes to map this data to, are likely required to determine the full collection of fungi across volunteers and samples. Because of the high abundance of bacterial DNA in stool samples, ITS2 sequencing may be both a more accurate and sensitive method for characterizing the human gut mycobiome, providing greater resolution compared to moderately deep WGS sequencing.

**Table 3:** Top 10 most prevalent fungi found in metagenomic WGS sequences

Species	Volunteers (*n* = 215)	Samples (*n* = 472)	Reads (*n* = 27,091,491,028)
*Malassezia restricta*	131	191	5829
*Saccharomyces cerevisiae*	128	198	6205
*Malassezia globosa*	115	168	2373
*Cyberlindnera jadinii*	67	92	88,922
*Saccharomyces pastorianus*	66	84	307
*Candida albicans*	45	55	2426
*Debaryomyces hansenii*	31	32	278
*Malassezia sympodialis*	24	28	92
*Alternaria alternata*	24	24	81
*Candida parapsilosis*	23	25	158

## Discussion

Previous studies have examined fungal communities largely in small disease centric cohorts, and information detailing the healthy human mycobiome in a large, well-studied cohort is lacking. In this study, extracted DNA from fecal samples from the Human Microbiome Project was used to investigate what constitutes a normal gut mycobiome. This study represents the first time the fecal mycobiome has been described in a large cohort of healthy individuals (over 100 volunteers), with longitudinal samples provided by each volunteer (up to three samples per volunteer, totaling 317 samples). Furthermore, this is the first study that includes ITS, 18S rRNA gene, 16S rRNA gene, and WGS metagenomic sequencing data on the same samples, thus enabling a validation of methods and correlative analyses. The results indicate that fungal diversity is lower than bacterial diversity in the gut, and that yeast genera such as *Saccharomyces*, *Malassezia*, and *Candida* are the most abundant genera present in this cohort. *Candida* spp. have commonly been identified as members of the healthy human mycobiome, not only in the gut [9, 20] but also at several other body sites, including the oral cavity [21, 22], vagina [24], and skin [23, 30]. Previous studies have observed high levels of *Malassezia* at different body sites, describing it as a prominent commensal of the skin and oral mycobiomes [21, 23]. Interestingly, a study by Hoffmann et al. examining the mycobiome of the gut in relation to diet in a smaller set of healthy volunteers recognized *Saccharomyces* and *Candida* as prevalent members of the gut mycobiome, but did not identify *Malassezia* as a member of the gut mycobiota [9]. The discrepancy between the Hoffmann study and the results in the current study are likely due to differences in study methodologies: while this study amplified the ITS2 region of the fungal rRNA operon, Hoffmann et al. amplified the Internal Transcribed Spacer 1 (ITS1) region. In data described in Additional file 4, amplification and sequencing of a fecal samples found that the primers used to amply the ITS1 region (ITS1F and ITS2 [31, 32], also used in the Hoffmann study) did not detect *Malassezia*, indicating that sequence mismatches in the primers may not allow for optimal amplification of *Malassezia* DNA. Alternatively, *Malassezia* may not have been identified in the Hoffmann study due to differences in cohort characteristics, such as diet or geographical location. While volunteers in this study were recruited from Houston, Texas, the volunteers in the Hoffmann study were recruited from Pennsylvania. Differences in climate may impact the fungi to which individuals are exposed, which may in turn impact the colonization of fungi in the gut.

We determined that the gut mycobiome is highly variable between individuals as well as within individuals over time. A similar trend was observed in a study following fungal communities in mice, where it was found that the gut mycobiome varied substantially over time in mice receiving antibiotics as well as untreated control mice [33]. Furthermore, it was observed that different cages of mice receiving the same treatment also varied in their dominant fungal lineage. These findings occurred in mice housed in the same animal facility and on a homogeneous diet. Additionally, a human gut mycobiome study comprised of 24 individuals with two sampling time points found that detection of the same fungus at both time points occurred less than 20% of the time [20]. While the gut mycobiome was found to be variable within individuals, others have shown that the oral mycobiome stays fairly stable over time within an individual [34]. These results prompt a fundamental unanswered question in the field: which, if any, fungi are truly colonizing the human gut? It is known that the human microbiome is greatly impacted by diet, environment, and lifestyle [9, 35–37]. However, a limitation to the current culture-independent techniques reported here is that they only assess DNA signatures. Thus, these data cannot distinguish between the DNA contributed from live or dead cells and do not differentiate microbes that are colonizing the gut from transients derived from our diet and/or environment. But culture-dependent studies have identified many of the same abundant fungi we have detected here, including *Candida* spp. [38–43], *Saccharomyces cerevisiae* [40, 43], *Malassezia* spp. [38, 39, 44], *Penicillium* spp. [38–40, 42], *Cladosporium* spp. [38, 42], and *Aspergillus* spp. [38–40, 42, 44]. *Candida*, *Penicillim*, and *Aspergillus* spp. have been identified in fecal samples from many different volunteers across several studies, but *Malassezia* and *Saccharomyces* spp. are cultured less consistently. *Malassezia* has more stringent growth conditions (i.e., it cannot be grown on common yeast-friendly medias like Sabouraud or Potato Dextrose), which could account for its lack of detection in many studies. *Saccharomyces*, on the other hand, is easily cultured, suggesting its high abundance and prevalence in ITS2 sequencing data may be originating from other sources, especially since it is a common component in many foods. This is also likely the case for *Cyberlindnera jadinii*, a food additive also known as “torula yeast,” which was found in high abundance in some volunteers. Mycologists Suhr and Hallen-Adams have proposed that the majority of fungal taxa detected in culture-independent studies are likely not viable in the gut due to growth constraints (e.g., several *Penicillium* species do not grow at 37 °C) or known ecological niches (e.g., *Ustilago maydis* is an obligate maize pathogen) [45]. Notwithstanding, colonization is not necessary to exert a biologically significant effect on the host (e.g., many proposed probiotics do not necessarily colonize the gut for prolonged periods [46, 47]). More research must be done to determine which fungi, if any, may be colonizing the human gut and how they may be impacting resident microbes and the host.

Comparing results between existing mycobiome studies presents many challenges. First, non-standardized approaches are used by various labs to explore the mycobiome, and analysis strategies are rapidly evolving. Many molecular and bioinformatics methods utilized by researchers were optimized for isolation and analysis of bacterial communities and may not always be appropriate for fungi. Although the extraction method used on HMP stool samples was optimized for bacterial community analysis, we determined that this did not have a significant effect on alpha diversity, beta diversity, or taxonomy compared to an extraction method utilizing harsher mechanical lysis that is similar to methods used in current mycobiome studies (Additional file 5). Furthermore, there is still debate on the optimal region of the rRNA operon to assay for fungal community profiling. While the ITS1 region is a common target for molecular studies, our laboratory and others have found ITS2 may be more suitable for detecting fungal commensals. A closer look at ITS1F and ITS2 primers revealed that these commonly used ITS1 region-targeting primers contain critical mismatches to common fungal taxa found in the human microbiome, including *Galactomyces geotrichum*, *Yarrowia lipolytica*, and fungi belonging to the Malasseziales and Tremellales orders [48]. Additionally, available fungal databases are quite sparse and less well-curated compared to bacterial databases, both in terms of the overall number of sequences and the accuracy of taxonomic information. Misidentifications in fungal databases occur frequently, a circumstance that is compounded by fungal dimorphism (the ability of some fungi to change morphologically between hyphal and yeast forms depending on environmental conditions). This phenomenon often results in different studies identifying identical ITS sequences as two different fungi. Moreover, database entries may contain insufficient taxonomic information to correctly identify fungi, leading to the “Fungi sp.” or “unclassified fungi” identifications seen in our and others’ data [20]. Our study found that approximately 17% of OTUs lacked taxonomic information. Finally, availability of fungal genomes is also lacking compared to bacteria, though there are efforts underway to change this [49]. This scarcity of complete fungal genomes makes identifying fungi in complex samples difficult and is compounded by the generally low relative abundance of fungi compared with other microbes. In the HMP samples used in this study, we found that fungal sequences constituted approximately 0.01% of the total number of metagenomic sequences. However, this number may increase as more fungal genomes are sequenced and more data may be mapped to these genomes.

To confirm that no major components of the mycobiome were being missed due to known ITS2 primer bias, a subset of samples were analyzed by broad eukaryotic 18S rRNA gene amplification and sequencing. Only one additional fungal genus, *Tritirachium*, was detected that was not among the named genera detected by ITS2 sequencing in the 89 shared samples. The discovery of this low abundance genus in a single sample was likely due to further sampling of a diverse sample rather than an ITS2 primer bias. The 18S rRNA gene results lend support to the completeness of the ITS2 fungal data but also demonstrate that fungi are not the only microeukaryotes present in the gut. In particular, the animal gut symbiont *Blastocystis* was present in 25% (11/44) of the volunteers examined, which is within the carriage range found in other developed countries. In contrast, *Dientamoeba fragilis*, another intestinal microeukaryote common in some healthy populations, was not detected in HMP samples [50]. The *Blastocystis* subtypes that were detected (ST1, ST2, ST3) are, together with ST4, the most frequently identified in humans [51]. Colonization by *Blastocystis* has been associated with increased bacterial diversity [52], and this held true for HMP samples. However, the detection of *Blastocystis* did not correspond to increased fungal diversity—yet another distinct attribute of the mycobiome. We also found that 18S rRNA gene sequencing data mapped to a variety of presumably dietary sources, such as fish, meat, fowl, and plants, raising the idea that perhaps 18S rRNA gene sequencing data could be used to validate, or as a surrogate for, dietary information collected by questionnaires.

## Conclusions

The human gut mycobiome is low in diversity compared to gut bacterial communities and is dominated by the yeast genera *Saccharomyces*, *Malassezia*, and *Candida*. Both inter- and intra-volunteer variability were high, yet several species tended to persist across all samples and within longitudinal samples belonging to a single individual. While no associations between the mycobiome and volunteer metadata were detected, correlation analysis revealed newly discovered relationships between and among bacterial and fungal taxa, and further studies of these correlations could identify novel means by which to modulate the abundance of specific microbiome constituents. Finally, 18S rRNA gene and WGS metagenomic sequencing aligned with the results of ITS2 sequencing, but ITS2 data provided greater resolution of the mycobiome membership, suggesting that ITS2 sequencing is a more accurate and sensitive method for studying the mycobiome in stool samples. Understanding what constitutes a “normal” or “healthy” gut mycobiome could assist in future research efforts to determine contributions of commensal fungi to the health of the host or the exacerbation of disease.

## Methods

### Sample collection and DNA extraction

Stool samples were collected and DNA was extracted as previously described [53]. In brief, stool samples were collected, and approximately 2 ml of stool was homogenized by vortexing in 5 ml of MO BIO lysis buffer (PowerLyzer PowerSoil Bead solution, MO BIO Laboratories). After slow-speed centrifugation, 1 ml of supernatant was added to MO BIO Garnet Bead tubes containing 750 μl of lysis buffer. The sample was then incubated at 65 °C for 10 min followed by 95 °C for 10 min. Further DNA extraction steps were performed using the standard protocol from the MO BIO PowerSoil DNA Isolation Kit. After initial sample extraction, aliquoted DNA samples were stored at − 80 °C before retrieval for this study. The present study only used DNA from stool samples collected at Baylor College of Medicine in Houston, TX, from volunteers who donated between one and three stool samples over the course of approximately 1 year. Detailed information about volunteer inclusion criteria, consent forms, sample collection, extraction protocols, and supplemental study information can be found on the HMP Data Analysis and Coordination Center website (http://www.hmpdacc.org/).

Harsher mechanical lysis methods are often employed for fungal DNA extraction because of the chitin in fungal cell walls [21, 38, 54, 55]. We wanted to confirm that the method used for microbial DNA extraction from HMP stool samples was not preventing us from capturing all the fungal diversity in the samples. Using stool from five healthy non-HMP donors, we compared fungal diversity and taxonomy using the HMP method of microbial DNA extraction (MO BIO PowerSoil DNA Isolation Kit) and a modified version of this protocol that is similar to what other investigators have used [21, 38, 54, 55]. This modified protocol included 0.5-mm glass beads in place of garnet beads and use of the FastPrep-24 Instrument (MP Biomedicals, speed 6.5 for 1 min, performed twice with a 5 min break in between) in place of a benchtop vortexer. Results (Additional file 5: Figure S2) revealed no difference in fungal taxonomy, alpha diversity, or beta diversity between the unmodified MO BIO PowerSoil DNA Isolation Kit protocol and the modified version in which harsher mechanical lysis steps were used.

### ITS2 amplification and sequencing

Before analyzing HMP samples, conditions for PCR were optimized as described in Additional file 4: Table S4 and S5. The Internal Transcribed Spacer 2 (ITS2) region was amplified from HMP stool DNA using primers ITS3 and ITS4 [32]. Each primer included an Illumina adapter and linker sequence designed using PrimerProspector [56]. Each reverse primer (ITS4) also contained a unique 12 base pair Golay barcode [57]. Amplification, sequencing, and index primers can be found in Additional file 6: Table S6. PCRs (20 μl total volume) contained 2 μl of Accuprime 10X PCR Buffer II (Invitrogen), 0.15 μl of Accuprime Taq High Fidelity DNA Polymerase (Invitrogen), 1 μl of each primer (0.4 μM final concentration), 14.25 μl of template DNA, and 1.60 μl of BSA. PCR cycling conditions were as follows: initial denaturation at 95 °C for 2 min, 35 amplification cycles of 95 °C for 20 s, 56 °C for 45 s, and 72 °C for 90 s, followed by a final extension step of 72 °C for 10 min. PCR products were visualized using agarose gel electrophoresis, quantified using Quant-iT PicoGreen dsDNA Assay Kit (Molecular Probes), and then cleaned using ChargeSwitch PCR Clean-Up Kit (Invitrogen). Samples were pooled and sequenced on the Illumina MiSeq platform using the Illumina MiSeq Reagent v3 600-cycle (2 × 300 bp) Kit.

### Bioinformatics and statistical analysis

#### ITS2 sequencing analysis

The ITS2 read pairs were demultiplexed based on the unique molecular barcodes. Reads were merged and filtered using USEARCH v7.0.1090 [58] using default settings, except with a minimum overlap length set to 50 bp and with staggered alignments enabled. A custom algorithm was used to cut off overhangs if read pairs were staggered. In the event of a conflict, the base with the higher Q score was chosen. Merged reads containing more than 0.5% expected errors were discarded.

ITS2 sequences were stepwise clustered into OTUs at a similarity cutoff value of 97% using the UPARSE pipeline [59]. Chimeras were removed using USEARCH v8.0.1517 and UCHIME [60]. OTUs were aligned against a combined database comprised of sequences from the NCBI GenBank Plant (including fungi) and Environmental databases [61]. Abundances were recovered by mapping the demultiplexed reads to the UPARSE OTUs. A custom script constructed an OTU table from the output files generated in the previous two steps. Unmapped (< 80% identity or < 95% coverage) OTUs were manually analyzed by BLASTN [62].

Samples were rarefied to 1954 reads/sample, unless otherwise noted, based on rarefaction analysis (Additional file 7: Figure S3a), to optimize number of sequences/sample without losing too many samples from the dataset. Analysis and visualization of microbiome communities was conducted in R version 3.3.3 [63], utilizing the phyloseq package version 1.19.1 [64] to import sample data and calculate alpha- and beta-diversity metrics. Plots were made using ggplot2 package version 2.2.1 [65], except for Fig. 4, which is described below. Significance of categorical variables was determined using the non-parametric Mann-Whitney test or Kruskal-Wallis test and adjusted for multiple comparisons with the FDR algorithm [66], unless otherwise stated. For box and whisker plots, the line represents the median value and the upper and lower hinges correspond to the first and third quartile. The whiskers extend from the box to the largest or smallest (upper or lower whisker, respectively) value no further than 1.5 * the inter-quartile range. Points plotted beyond the whiskers are considered outliers.

To determine associations with host phenotype, we performed the “EnvFit” function within the “Vegan” package version 2.4-2 [25] in R to determine covariates significantly associated with the mycobiome profiles. The model was performed based on the Bray-Curtis dissimilarity in NMDS ordination. Significance was determined by 10,000 permutations, and resulting *p* values were adjusted for multiple comparisons with the FDR algorithm [66].

Correlation analysis between taxa was performed using SparCC [26]. Fungal and bacterial taxa must make up at least 0.05% of the overall abundance to be included in the correlation analysis. Correlation values were plotted in R using the corrplot package version 0.77 [67]. *p* values were adjusted for multiple comparisons with the FDR algorithm [66]. Significant correlation values are signified by a colored square (either blue or red). That is, squares that lack color represent correlation values that are not significant (*p* > 0.05 after FDR adjustment) based on the statistical test built into the package. For comparisons between bacterial and fungal taxa, samples were included only if they had both 16S rRNA gene sequencing and ITS2 sequencing data available. Raw correlation values and *p* values for SparCC analyses can be found in Additional file 8: Tables S8, S9, S10, S11, S12, and S13.

#### 16S rRNA gene sequencing analysis

HMP 16S V3-V5 sequences were downloaded from http://www.hmpdacc.org. Read pairs were demultiplexed and then merged using USEARCH v7.0.1090 [58], allowing zero mismatches and a minimum overlap of 50 bp. Sequences were clustered into OTUs at a similarity cutoff value of 97% using the UPARSE algorithm [59]. OTUs were subsequently mapped to the SILVA database v123 [68] for taxonomic classification. An OTU table was constructed and used for further analyses.

#### 18S rRNA gene analysis

The eukaryotic 18S rRNA gene was amplified, sequenced, and processed as for ITS2, with the following exceptions: (1) 5 μl template was used in PCR, with annealing at 50 °C, (2) the only reads retained were those that contained no mismatches to expected barcode/linker/primer sequences, (3) to maximize phylogeny resolution, sequences were stepwise clustered into OTUs at a similarity cutoff value of 99%, and (4) OTUs were mapped to the SILVA database [68]. Dual-barcoded primers were composed of the 58 bp Illumina flow cell binding and sequencing primer regions, one of 22 different 4 bp barcodes, a TT linker, and the 20 or 23 bp 18S rRNA gene-targeting sequence (Additional file 6: Table S7).

Differences in bacterial and fungal diversity associated with the presence of *Blastocystis* were determined using the Mann-Whitney test with one random sample/volunteer. There was 16S rRNA gene sequencing data for 5 *Blastocystis*-positive and 26 *Blastocystis*-negative volunteers and ITS2 sequencing data for 11 *Blastocystis*-positive and 35 *Blastocystis*-negative volunteers. Rarefaction curves for 18S rRNA gene sequencing data is provided in Additional file 7: Figure S3b.

#### Fungi in metagenomic sequences

The HMP HiSeq metagenomic data set (from NCBI Accession PRJNA43017, also available at http://www.hmpdacc.org/) consisted of 27,091,491,028 total sequences from 472 stool samples of 215 volunteers, 64 of which overlapped with volunteers whose samples underwent ITS2 sequencing in this study. MetaPhlAn2 [69] and searches against ITS databases yielded very few fungal hits. Therefore, we instead mapped reads against all 1315 fungal genomes in NCBI (downloaded July 19, 2016). However, all examined hits initially mapped to either bacterial contamination within the fungal data, or fungal genes with high identity to bacterial homologs. Therefore, we used a 35 bp seed size MegaBLAST of the fungal genome assemblies against all NCBI genome assemblies to eliminate non-fungal hits of ≥ 85% identity, reducing the database of fungal genomes 1 gigabase (GB) from its original 47 GB size (46 GB final size). HMP reads were trimmed using BBDuk and searched against the cleaned fungal database using Bowtie2 [70] with a seed size of 20 bp. Positive hits were further refined by (1) only examining hits with 0 or 1 mismatches, (2) searching the reads against Silva SSU and LSU v. 128 databases [68] using Bowtie2 and removing any hits, and (3) running all translated and untranslated reads against non-redundant GenBank protein and nucleotide databases using DIAMOND [71] and BLASTN [72] to remove those reads that hit bacteria, archaea, non-fungal eukaryotes, or nothing. Taxonomic names were assigned from the top nucleotide (96%) or protein (4% with no taxonomic name by BLASTN) hit. Further pipeline development is needed to better classify reads from conserved fungal genes that match multiple taxa equally well. Each taxonomic name was assigned from the top hit to the fungal genome assembly database; however, if multiple taxa hit equally, the assigned name was randomly chosen. Therefore, conserved fungal genes have the potential to overestimate the contribution of closely related taxa.

## Additional files

Additional file 1: List of metadata tested for associations with mycobiome. (PDF 17 kb)

Additional file 2: *Blastocystis*. Description – **Table S1:** *Blastocystis*-positive samples, percentage of 18S sequences *Blastocystis*, and subtypes of *Blastocystis* identified. **Figure S1:** Alpha diversity of samples in which *Blastocystis* was (blue) or was not (yellow) detected. a 16S rRNA gene alpha diversity from dataset rarefied to 735 reads. Due to small sample size, the statistical significance varied greatly depending on the rarefaction and randomly chosen samples. b ITS2 alpha diversity from dataset rarefied to 4043 reads. (PDF 51 kb)

Additional file 3: Fungi in metagenomic sequences. Description – **Table S2:** Complete list of fungi found in metagenomic sequencing data. **Table S3:** Fungal reads found in metagenomic sequencing data. (XLSX 6137 kb)

Additional file 4: Comparative analysis of fungal primers. Description – PCR primer optimization background, methods, and results. **Table S4:** Primers used in PCR primer optimization tests. **Table S5:** Alpha Diversity of different primer pairs. (PDF 201 kb)

Additional file 5: Extraction methods comparison. Description – Fungal DNA extraction methods comparison methods and results. **Figure S2:** a Alpha diversity (Observed OTUs and Shannon diversity) of both fungal DNA extraction methods. b Beta diversity (Bray-Curtis dissimilarity) of samples, colored by method, shaped by donor. c Relative abundance of fungal taxa. (PDF 344 kb)

Additional file 6: ITS2 and 18S gene HMP study primers. Description – **Table S6:** ITS2 sequencing and index primers used in this study. **Table S7:** 18S sequencing primers used in this study. (XLSX 24 kb)

Additional file 7: Rarefaction curves for ITS2 and 18S rRNA gene sequencing. Description – **Figure S3:** Rarefaction analysis curves for a ITS2 sequencing data and b 18S rRNA gene sequencing data. Shaded region represents 95% confidence interval. (PDF 29 kb)

Additional file 8: SparCC correlations and *p*-values. Description – **Table S8:** ITS2 vs ITS2 correlations. **Table S9:** ITS2 vs ITS2 *p*-values. **Table S10:** ITS2 vs ITS2 FDR-adjusted *p*-values. **Table S11:** ITS2 vs 16S correlations. **Table S12:** ITS2 vs 16S *p*-values. **Table S13:** ITS2 vs 16S FDR-adjusted *p*-values. (XLSX 282 kb)

## Abbreviations

GB: Gigabase
HMP: Human Microbiome Project
ITS: Internal Transcribed Spacer
OTU: Operational taxonomic unit
PCoA: Principal coordinates analysis
ST: Subtype
WGS: Whole genome shotgun
