# Supplementary material for: The gut mycobiome of the Human Microbiome Project healthy cohort
Source: Microbiome. 2017 Nov 25;5:153. doi: 10.1186/s40168-017-0373-4 (PMC5702186; doi:10.1186/s40168-017-0373-4)
Supplement: Supplementary file 1 — List of metadata tested for associations with mycobiome. (PDF 17 kb) [file 40168_2017_373_MOESM1_ESM.pdf]

List of metadata tested for correlations with mycobiome.

- Education level
- Health insurance coverage
- Dental insurance coverage
- Occupation
- Tobacco use and type
- Body temperature
- Systolic and diastolic blood pressure
- Pulse rate
- Weight
- Height
- BMI
- Gender
- Age
- Ethnicity/race
- Birth country
- Mother's birth country
- Father's birth contry
- Diet (3 options – meat/fish/poultry at least 3 days/week, meat/fish/poultry 1-2 days/week, eggs/cheese/other dairy products but no meat/fish/poultry)
- Breast fed as child
- Given birth and delivery mode if answered yes
- Medication (examples – antacids, antidepressants, contraceptives, vitamins/supplements, etc.)
