# Supplementary material for: The gut mycobiome of the Human Microbiome Project healthy cohort
Source: Microbiome. 2017 Nov 25;5:153. doi: 10.1186/s40168-017-0373-4 (PMC5702186; doi:10.1186/s40168-017-0373-4)
Supplement: Supplementary file 2 — Blastocystis. Description – Table S1: Blastocystis-positive samples, percentage of 18S sequences Blastocystis, and subtypes of Blastocystis identified. Figure S1: Alpha diversity of samples in which Blastocystis was (blue) or was not (yellow) detected. a 16S rRNA gene alpha diversity from dataset rarefied to 735 reads. Due to small sample size, the statistical significance varied greatly depending on the rarefaction and randomly chosen samples. b ITS2 alpha diversity from dataset rarefied to 4043 reads. (PDF 51 kb) [file 40168_2017_373_MOESM2_ESM.pdf]

Table S1
Blastocystis-positive samples, percentage of 18S sequences *Blastocystis*, and subtypes of *Blastocystis* identified.

**HMP Sample**	**% 18S sequences *Blastocystis***	**Subtype**
Vol.241676.3	21.15%	ST3
Vol.241710.1	100.0%	ST2
Vol.241710.2	100.0%	ST2
Vol.241711.1	100.0%	ST1
Vol.241711.2	100.0%	ST1
Vol.241711.3	100.0%	ST1
Vol.241712.1	68.2%	ST3
Vol.241721.3	0.2%	ST2
Vol.241754.1	100.0%	ST3
Vol.241754.2	100.0%	ST3
Vol.241754.3	100.0%	ST1
Vol.241755.3	0.03%	ST1
Vol.241776.3	74.6%	ST3
Vol.241780.2	100.0%	ST3
Vol.241780.3	100.0%	ST3
Vol.241849.1	93.1%	ST3
Vol.241849.2	99.9%	ST3
Vol.241849.3	100.0%	ST3
Vol.285223.1	3.3%	ST1

Figure S1

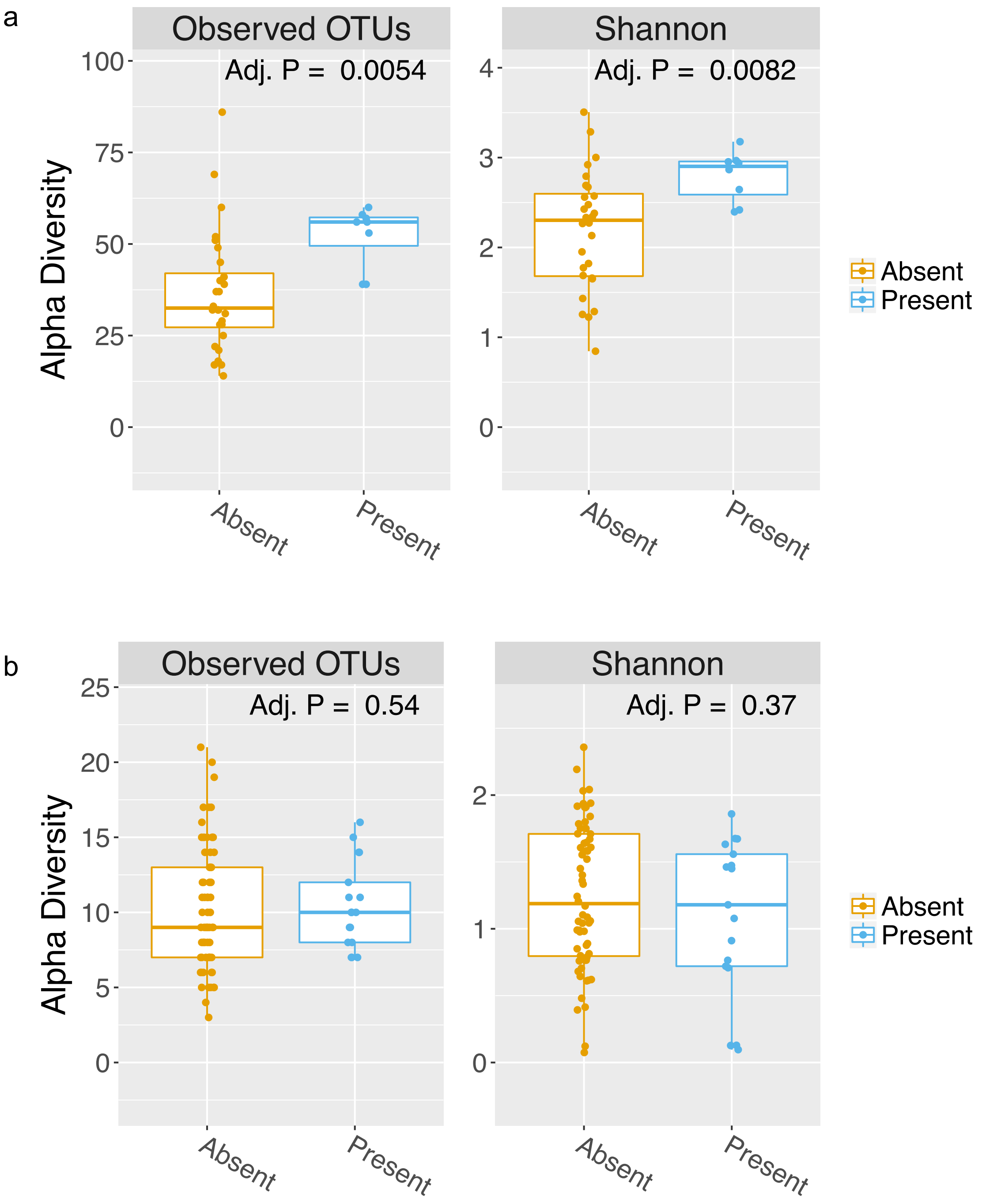

Figure S1: Alpha diversity of samples in which *Blastocystis* was (blue) or was not (yellow) detected. **a** 16S rRNA gene alpha diversity from dataset rarefied to 735 reads. Due to small sample size, the statistical significance varied greatly depending on the rarefaction and randomly chosen samples. **b** ITS2 alpha diversity from dataset rarefied to 4043 reads.
