# Supplementary material for: The gut mycobiome of the Human Microbiome Project healthy cohort
Source: Microbiome. 2017 Nov 25;5:153. doi: 10.1186/s40168-017-0373-4 (PMC5702186; doi:10.1186/s40168-017-0373-4)
Supplement: Supplementary file 4 — Comparative analysis of fungal primers. Description – PCR primer optimization background, methods, and results. Table S4: Primers used in PCR primer optimization tests. Table S5: Alpha Diversity of different primer pairs. (PDF 201 kb) [file 40168_2017_373_MOESM4_ESM.pdf]

Comparative Analysis of Fungal Primers

Background

Most eukaryotes encode ribosomal genes in an operon, with a relatively unconserved internal transcribed spacer (ITS) between conserved genes (order = 18S - ITS1 - 5.8S - ITS2 - 28S). The ITS region is widely recognized as the best available single region to examine for comparative fungal analysis[1]. Many different primer sets have been used to amplify fungal ITS1, ITS2, or the entire span containing both ITS regions. Although ideally both the ITS1 and ITS2 regions would be amplified and sequenced (suggested by Bellemain et al.[2]), it is not practical for screening large numbers of samples using current MiSeq technology.

Optimal ITS primers take into account several criteria: 1) *taxonomic coverage*, amplifying as broad a range of fungi as possible, 2) *taxonomic discrimination*, not amplifying other groups, such as the numerically dominant bacteria, digested plants, or shed host human cells in fecal samples, 3) *taxonomic resolution* provided by the amplicon sequences, 4) *amplicon length* suitable for sequencing method (here an Illumina MiSeq 2 x 300), and 5) *amplification efficiency.* Since ITS1 and ITS2-specific amplicons have comparable taxonomic discrimination, taxonomic resolution, and lengths, when deciding which region to target, the major concerns were which primers had the best amplification efficiency, and the broadest and least biased taxonomic coverage.

Since it was not practical to test very many primer sets, we chose to test those commonly used to target ITS1 (ITS1 and ITS2[3], here referred to as ITS1F and ITS2R) and ITS2 (ITS3 and ITS4[3], here referred to as ITS3F and ITS4R) (primer names and sequences listed **Table S4**). We also tested the reported winner of a fungal primer set comparison, ITS2-targeting ITS86F and ITS4R[4, 5], and as a control, 18S rRNA gene-targeting 775F-1121R (*Saccharomyces cerevisiae* numbering). These 18S rRNA gene primers were designed to target the broadest range of fungi possible, while excluding archaea, bacteria, plants, and animals that are also found in the human gut. Though the 18S rRNA gene amplicon may have better taxonomic coverage, it has less heterogeneity and therefore less taxonomic discrimination than ITS regions, making it less attractive for screening samples.

In silico testing using databases such as UNITE[6] & Silva[7] can yield incorrectly low estimates of the primer coverage because the ends of 18S and 28S rRNA genes (where primers anneal) are rarely amplified and therefore rarely present in the databases. However, for testing primers that anneal to part of the 5.8S rRNA gene, UNITE is useful. ITS2R = ITS3F (reverse complement of each other) and ITS86F match 93% and 96%, respectively, of fungi in the 99% OTU UNITE version 6 release 9/10/2014 dataset (using standard threshold in PrimerProspector[8]). Although several studies have endeavored to establish an optimal primer set for ecological studies (e.g., [2, 4, 9-11]), ultimately the best choice will depend on the composition of the targeted community. To our knowledge there has not previously been such a comparison with fecal samples.

Methods

The DNA of five young children's fecal samples, five adult fecal samples, and a mock community were used as template in PCR to compare the amplification efficiency and taxonomic coverage of four fungal-targeting primer sets. The mock community was composed of equivalent parts genomic DNA of *Trametes versicolor*, *Cladosporium sp.*, *Penicillium solitum*, *Penicillium sp.*, *Saccharomyces boulardii*, *Aspergillus fumigatus*, and *Cryptococcus neoformans*. AccuPrime Taq HighFidelity and buffer (Life Technologies), template DNA, and 600 nM barcoded primers (Integrated DNA Technologies; Supplemental Table 1) were used in PCR at 94°C 30 s, then 35 cycles of [94°C 30s; 48°C (ITS86F-ITS4R) or 57°C (all others) 30 s; 68°C 1 min (including 30 s excess to reduce selection for small amplicons)]. The annealing temperatures were chosen based on amplification of ascomycete, basidiomycete, plant, animal, and bacterial controls at a range of temperatures as measured by qPCR.

When the 11 PCR-amplified samples were run on an agarose gel, there was a detectable band from 9/11 for 775F-1121R, 5/11 for ITS1F-ITS2R, and 8/11 for ITS3F-ITS4R and ITS86F-ITS4R. A reduced amplification efficiency from the ITS1 primer set was also observed when, in another experiment, various environmental and cultured isolates were screened: 31/49 (ITS1F-ITS2R) vs. 37/49 (ITS3F-ITS4R) had gel-detectable bands. The reduced amplification efficiency for ITS1 was also reflected in the number of sequence reads, with that primer set having the lowest for 5/6 samples (**Table S5**). Also, though it has been suggested that ITS1 is preferable to ITS2 due to less of a predicted size difference between Ascomycetes and Basidiomycota[9], and therefore theoretically less amplification bias toward the generally smaller Ascomycetes, we found ITS2 amplicons were of a more consistent size. This finding has also been found by others (e.g., [12]).

The two different sets of five amplicons (child and adult samples) with the same barcodes were unintentionally pooled together, so results reflect collective diversity. Sequencing was done on an Illumina MiSeq using the 2 x 300 kit (as described in main methods), resulting in a median of 11,000 merged reads. To calculate diversity, the dataset for each sample was rarefied to the maximum number of reads that would allow analysis of all four primer sets. As shown in the table below (**Table S5**) ITS1F-ITS2R had the greatest diversity twice (samples 1 & 2), both ITS2 primer sets had greater diversity than ITS1F-ITS2R once (in sample 5), while the other two samples had different winners, depending on the diversity measure used. The ITS3F-ITS4R and ITS86F-ITS4R results were very similar. ITS86F-ITS4R had greater diversity than ITS3F-ITS4R in three samples (observed OTUs and Inverse Simpson), ITS3F-ITS4R had greater diversity than ITS86F-ITS4R in one (observed OTUs) or two (Inverse Simpson) samples, and for one (Inverse Simpson) or two (observed OTUs) samples they had the same diversity.

Looking at taxonomy, ITS1F-ITS2R missed *Malassezia,* while ITS3F-ITS4R and ITS86F-ITS4R missed Sporidiobolales/*Rhodotorula* (not present at all, while detected in ≥ 2 samples with the other primer sets). Surprisingly no fungal groups were exclusively found with 775F-1121R, likely because only Ascomycota and Basidiomycota were present, and it is the amplification of fungal groups outside these phyla that 18S rRNA gene primers are superior. Looking at the mock community, the 18S rRNA gene and ITS2 primer sets found the appropriate six OTUs. However, ITS1F-ITS2R missed *Cryptococcus neoformans* and *Aspergillus fumigatus* completely. The ITS1F-ITS2R

amplification of mock community also likely overestimated the abundance of *Trametes versicolor* (91% of the reads).

Table S4: Primers used in PCR primer optimization tests.

Primer name	Illumina Adapter, Illumina sequencing primer target, Barcode, Amplicon targeting region
Illumina18S.775F.1	CAAGCAGAAGACGGCATACGAGATGTGACTGGAGTTCAGACGTGT GCTCTTCCGATCTTAATGARRAAATTAGAGTGTTCAAAGCAG
Illumina18S.775F.2	CAAGCAGAAGACGGCATACGAGATGTGACTGGAGTTCAGACGTGT GCTCTTCCGATCTCGTTGARRAAATTAGAGTGTTCAAAGCAG
Illumina18S.775F.3	CAAGCAGAAGACGGCATACGAGATGTGACTGGAGTTCAGACGTGT GCTCTTCCGATCTATGTGARRAAATTAGAGTGTTCAAAGCAG
Illumina18S.1121R.1	AATGATACGGCGACCACCGAGATCTACACTCTTTCCCTACACGAC GCTCTTCCGATCTCCTAAACCTTYAAGTTTCAGCCTTGCG
Illumina18S.1121R.2	AATGATACGGCGACCACCGAGATCTACACTCTTTCCCTACACGAC GCTCTTCCGATCTCTACGGCCTTYAAGTTTCAGCCTTGCG
IlluminaITS1F.1	CAAGCAGAAGACGGCATACGAGATGTGACTGGAGTTCAGACGTGT GCTCTTCCGATCTTGCAGACTTGGTCATTTAGAGGAAGTAA
IlluminaITS1F.2	CAAGCAGAAGACGGCATACGAGATGTGACTGGAGTTCAGACGTGT GCTCTTCCGATCTTCCAACCTTGGTCATTTAGAGGAAGTAA
IlluminaITS1F.3	CAAGCAGAAGACGGCATACGAGATGTGACTGGAGTTCAGACGTGT GCTCTTCCGATCTCCATCACTTGGTCATTTAGAGGAAGTAA
IlluminaITS2R.1	AATGATACGGCGACCACCGAGATCTACACTCTTTCCCTACACGAC GCTCTTCCGATCTCATAGGGCTGCGTTCTTCATCGATGC
IlluminaITS2R.2	AATGATACGGCGACCACCGAGATCTACACTCTTTCCCTACACGAC GCTCTTCCGATCTGTGGTAGCTGCGTTCTTCATCGATGC
IlluminaITS86F.1	CAAGCAGAAGACGGCATACGAGATGTGACTGGAGTTCAGACGTGT GCTCTTCCGATCTTGGGAGGTGAATCATCGAATCTTTGAA
IlluminaITS86F.2	CAAGCAGAAGACGGCATACGAGATGTGACTGGAGTTCAGACGTGT GCTCTTCCGATCTACTTTAGTGAATCATCGAATCTTTGAA
IlluminaITS86F.3	CAAGCAGAAGACGGCATACGAGATGTGACTGGAGTTCAGACGTGT GCTCTTCCGATCTAGGGTGGTGAATCATCGAATCTTTGAA
IlluminaITS4R.1	AATGATACGGCGACCACCGAGATCTACACTCTTTCCCTACACGAC GCTCTTCCGATCTGAGCAATCCTCCGCTTATTGATATGC
IlluminaITS4R.2	AATGATACGGCGACCACCGAGATCTACACTCTTTCCCTACACGAC GCTCTTCCGATCTCATCCTTCCTCCGCTTATTGATATGC
IlluminaITS3F.1	CAAGCAGAAGACGGCATACGAGATGTGACTGGAGTTCAGACGTGT GCTCTTCCGATCTTGTTGCGCATCGATGAAGAACGCAGC
IlluminaITS3F.2	CAAGCAGAAGACGGCATACGAGATGTGACTGGAGTTCAGACGTGT GCTCTTCCGATCTGTTTCTGCATCGATGAAGAACGCAGC
IlluminaITS3F.3	CAAGCAGAAGACGGCATACGAGATGTGACTGGAGTTCAGACGTGT GCTCTTCCGATCTATGTCCGCATCGATGAAGAACGCAGC

Table S5: Alpha Diversity of different primer pairs.
Observed OTUs (Inverse Simpson)

Rarefaction[a]	Samples	775F-1121R	ITS1F-ITS2R	ITS3F-ITS4R	ITS86F-ITS4R
1684	Child/Adult 1	11 (1.2)	**17 (7.3)**[b]	8 (1.1)	12 (1.2)
153	Child/Adult 2	2 (1.2)	**9 (2.0)**	2 (1.6)	2 (1.5)
1960	Child/Adult 3	8 (1.6)	8 (2.0)	12 (1.5)	**13 (2.1)**
8129	Child/Adult 4	17 (**4.0**)	30 (2.7)	**43** (2.6)	42 (2.2)
562	Child/Adult 5	11 (1.2)	25 (4.1)	29 (6.2)	**41 (7.0)**
6305	Mock	**6 (2.5)**	4 (1.2)	**6 (2.5)**	**6 (2.5)**

[a] The dataset for each sample was rarefied to the maximum number of reads that would allow analysis of all four primer sets. Non-fungal taxonomic assignments (~2% of total) were removed from analysis.
[b] The highest diversity values are in bold.

Summary

ITS1F-ITS2R had the lowest amplification efficiency, lowest number of sequence reads (in 5 of 6 combined fecal samples), incomplete detection of mock community representatives (4/6), and amplicon diversity roughly equivalent to the ITS2 primer sets. An ITS2 primer set would therefore be preferable. However, there was no clear winner between ITS3F and ITS86F, since their amplification efficiencies and taxonomic coverage were similar.

References

1. Schoch CL, Seifert KA, Huhndorf S, Robert V, Spouge JL, Levesque CA, Chen W, Fungal Barcoding C, Fungal Barcoding Consortium Author L. Nuclear ribosomal internal transcribed spacer (ITS) region as a universal DNA barcode marker for Fungi. Proc Natl Acad Sci U S A. 2012; 109:6241-6246.
2. Bellemain E, Carlsen T, Brochmann C, Coissac E, Taberlet P, Kauserud H. ITS as an environmental DNA barcode for fungi: an in silico approach reveals potential PCR biases. BMC Microbiol. 2010; 10:189.
3. Kasai K, Nakamura Y, White R. Amplification of a variable number of tandem repeats (VNTR) locus (pMCT118) by the polymerase chain reaction (PCR) and its application to forensic science. J Forensic Sci. 1990; 35:1196-1200.
4. Op De Beeck M, Lievens B, Busschaert P, Declerck S, Vangronsveld J, Colpaert JV. Comparison and validation of some ITS primer pairs useful for fungal metabarcoding studies. PLoS One. 2014; 9:e97629.
5. Vancov T, Keen B. Amplification of soil fungal community DNA using the ITS86F and ITS4 primers. FEMS Microbiol Lett. 2009; 296:91-96.
6. Koljalg U, Nilsson RH, Abarenkov K, Tedersoo L, Taylor AF, Bahram M, Bates ST, Bruns TD, Bengtsson-Palme J, Callaghan TM, et al. Towards a unified paradigm for sequence-based identification of fungi. Mol Ecol. 2013; 22:5271-5277.
7. Quast C, Pruesse E, Yilmaz P, Gerken J, Schweer T, Yarza P, Peplies J, Glockner FO. The SILVA ribosomal RNA gene database project: improved data processing and web-based tools. Nucleic Acids Res. 2013; 41:D590-596.

8. Walters WA, Caporaso JG, Lauber CL, Berg-Lyons D, Fierer N, Knight R. PrimerProspector: de novo design and taxonomic analysis of barcoded polymerase chain reaction primers. Bioinformatics. 2011; 27:1159-1161.
9. Bokulich NA, Mills DA. Improved selection of internal transcribed spacer-specific primers enables quantitative, ultra-high-throughput profiling of fungal communities. Appl Environ Microbiol. 2013; 79:2519-2526.
10. Toju H, Tanabe AS, Yamamoto S, Sato H. High-coverage ITS primers for the DNA-based identification of ascomycetes and basidiomycetes in environmental samples. PLoS One. 2012; 7:e40863.
11. Hadziavdic K, Lekang K, Lanzen A, Jonassen I, Thompson EM, Troedsson C. Characterization of the 18S rRNA gene for designing universal eukaryote specific primers. PLoS One. 2014; 9:e87624.
12. Lindahl BD, Nilsson RH, Tedersoo L, Abarenkov K, Carlsen T, Kjoller R, Koljalg U, Pennanen T, Rosendahl S, Stenlid J, Kauserud H. Fungal community analysis by high-throughput sequencing of amplified markers--a user's guide. New Phytol. 2013; 199:288-299.
