# Supplementary material for: The gut mycobiome of the Human Microbiome Project healthy cohort
Source: Microbiome. 2017 Nov 25;5:153. doi: 10.1186/s40168-017-0373-4 (PMC5702186; doi:10.1186/s40168-017-0373-4)
Supplement: Supplementary file 5 — Extraction methods comparison. Description – Fungal DNA extraction methods comparison methods and results. Figure S2: a Alpha diversity (Observed OTUs and Shannon diversity) of both fungal DNA extraction methods. b Beta diversity (Bray-Curtis dissimilarity) of samples, colored by method, shaped by donor. c Relative abundance of fungal taxa. (PDF 344 kb) [file 40168_2017_373_MOESM5_ESM.pdf]

Additional file 4. Extraction methods comparison

DNA extraction was performed by two different methods: 1. MO BIO PowerSoil DNA Isolation Kit, denoted “PowerSoil” in figures, (MO BIO Laboratories, Cat # 12888-50) using manufacturer’s suggested protocol, and 2. the same kit, with a modified lysis step, denoted “Fungal” in figures. Briefly, instead of using garnet bead tubes provided in the kit, alternative bead tubes were used containing .5 mm glass beads (MO BIO Laboratories, Cat # 13116-50) with 500 μl of Bead Solution (MO BIO Laboratories, Cat # 12855-50-BS) . Also, instead of vortexing bead tubes for 10 minutes using a vortex adapter, bead tubes were placed in the FastPrep-24 Instrument (MP-Biomedicals) at level 6.5 for 1 min (performed twice, with a 5 minute resting time in between). After mechanical lysis, manufacturer’s suggested protocol was followed for PowerSoil DNA Isolation kit.

Five stool samples from healthy donors (non-HMP donors) were extracted in triplicate. DNA was amplified and sequenced using the ITS2 protocol described in the methods section of this paper. Samples were rarefied to 2,200 reads/sample, which resulted in one sample being removed due to low number of reads.

We found no significant difference in alpha diversity (**Figure S2a**), beta diversity (**Figure S2b**) or taxonomy (**Figure S2c**) between the two methods.

Figure S2

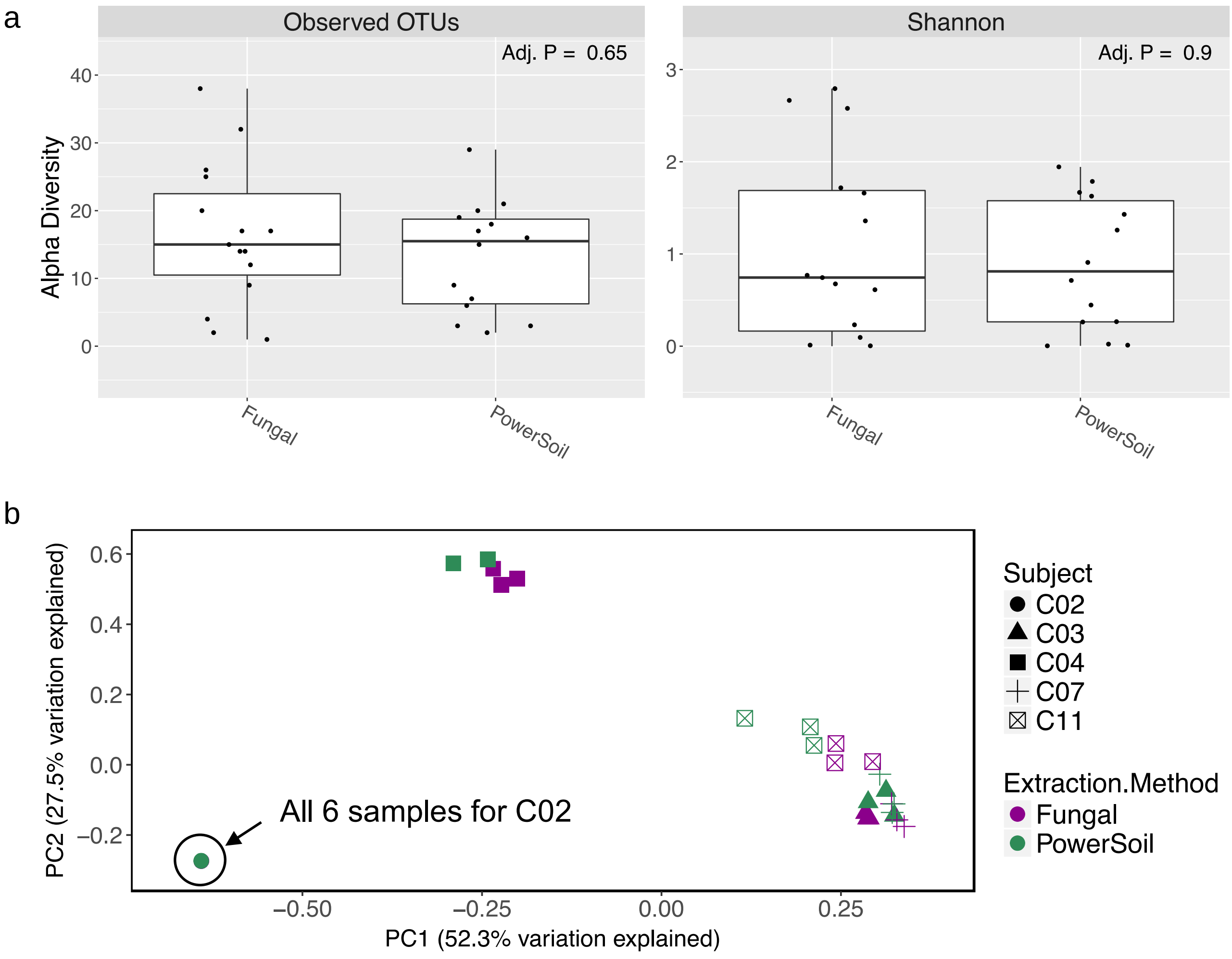

c

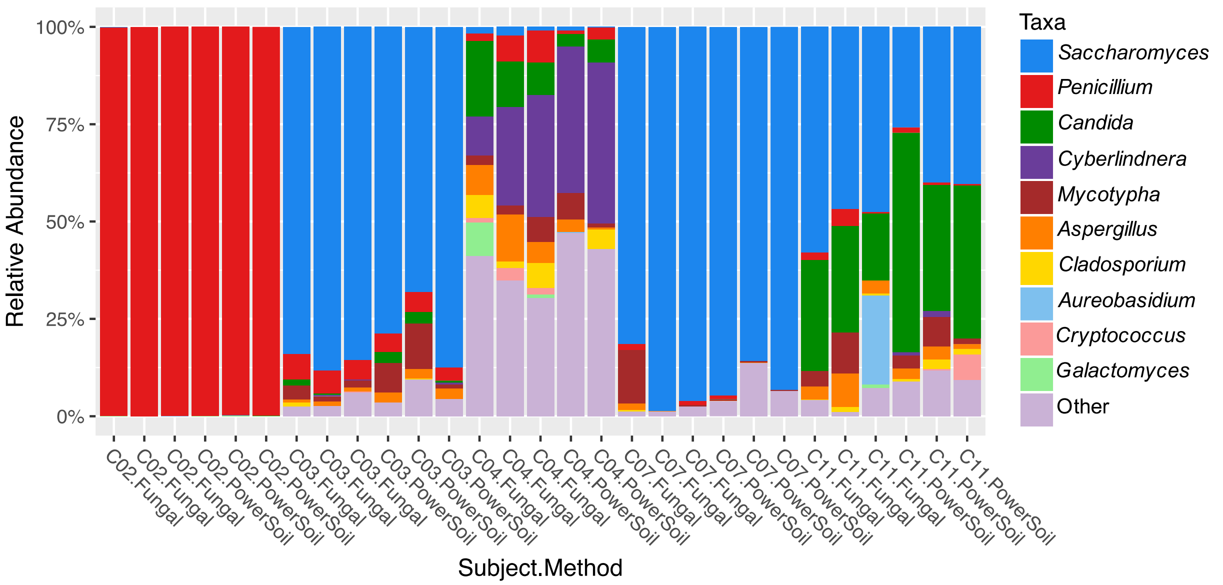

Figure S2: **a** Alpha diversity (Observed OTUs and Shannon diversity) of both fungal DNA extraction methods. **b** Principal coordinates analysis plot showing beta diversity (Bray Curtis Dissimilarity) of samples, colored by method, shaped by donor. **c** Relative abundance of fungal taxa.
