# Supplementary figures and images for: The gut mycobiome of the Human Microbiome Project healthy cohort
Source: Microbiome. 2017 Nov 25;5:153. doi: 10.1186/s40168-017-0373-4 (PMC5702186; doi:10.1186/s40168-017-0373-4)

**a**

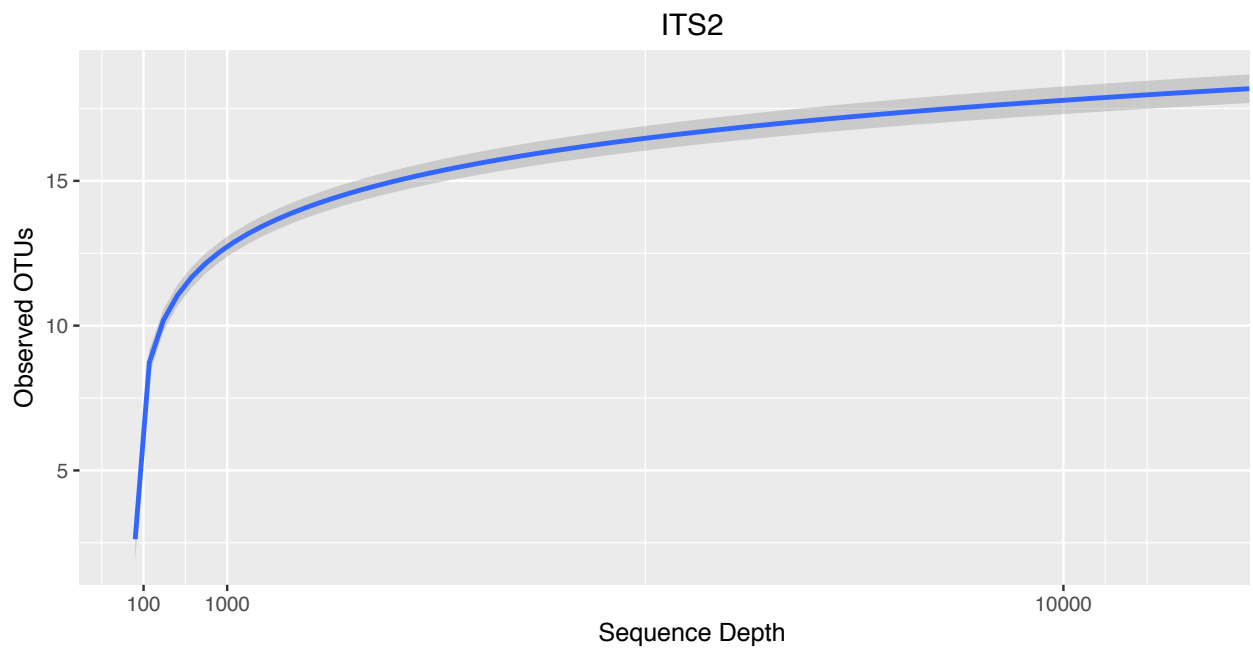

**b**

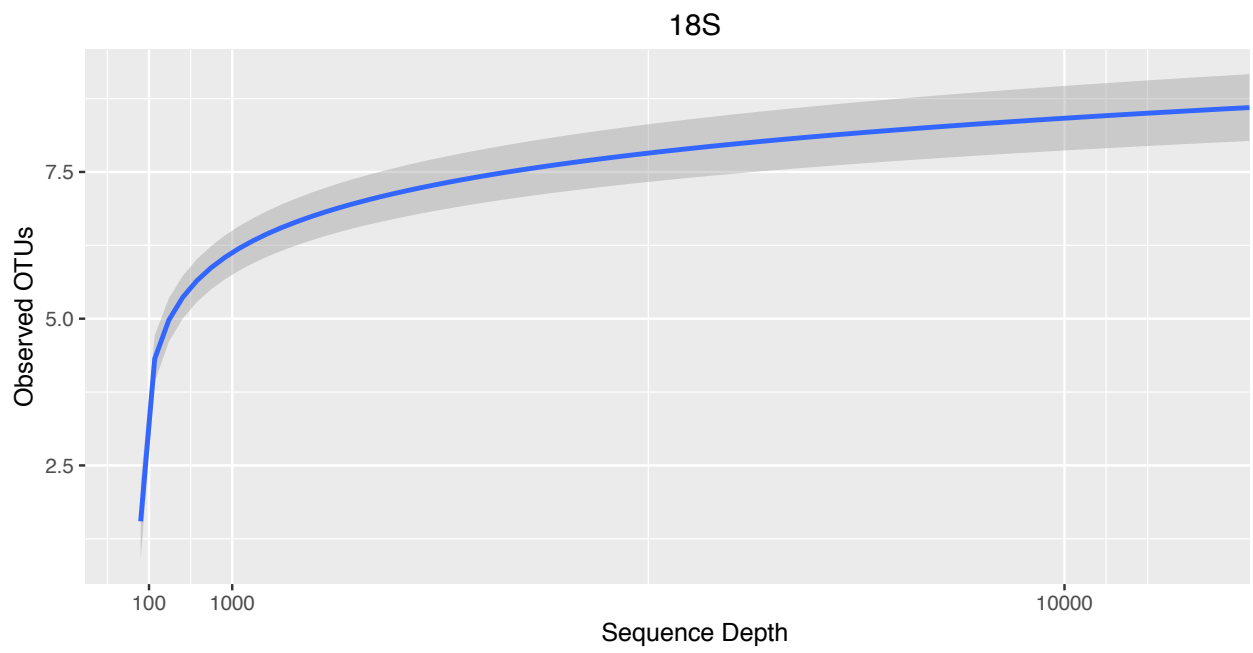

Supplement: Supplementary file 7 — Rarefaction curves for ITS2 and 18S rRNA gene sequencing. Description – Figure S3: Rarefaction analysis curves for a ITS2 sequencing data and b 18S rRNA gene sequencing data. Shaded region represents 95% confidence interval. (PDF 29 kb) [file 40168_2017_373_MOESM7_ESM.pdf]
